# Supplementary material for: Investigation of the Genetic Architecture of Pigs Subjected to Breeding Intensification
Source: Genes (Basel). 2022 Jan 22;13(2):197. doi: 10.3390/genes13020197 (PMC8871947; doi:10.3390/genes13020197)
Supplement: Supplementary file 1 [file genes-13-00197-s001.zip › genes-1550171-Table S2.pdf]

Table S2. Summary statistics of realized autozygosity in each HBD class (as a percentage of genome).

		mean	sd	min	max	range	se
R_4	LW_A	0,0043	0,0148	0,0000	0,0725	0,0725	0,0029
	LW_B	0,0050	0,0149	0,0000	0,0697	0,0697	0,0028
R_8	LW_A	0,0127	0,0216	0,0000	0,0839	0,0839	0,0042
	LW_B	0,0275	0,0346	0,0000	0,1078	0,1078	0,0064
R_16	LW_A	0,0329	0,0273	0,0000	0,0813	0,0813	0,0054
	LW_B	0,0557	0,0536	0,0000	0,1464	0,1464	0,0100
R_32	LW_A	0,0304	0,0260	0,0000	0,0814	0,0814	0,0051
	LW_B	0,0587	0,0552	0,0000	0,1808	0,1808	0,0103
R_64	LW_A	0,0159	0,0297	0,0000	0,0909	0,0909	0,0058
	LW_B	0,0386	0,0470	0,0000	0,1427	0,1427	0,0087
R_128	LW_A	0,0529	0,0350	0,0000	0,1328	0,1328	0,0069
	LW_B	0,0968	0,0363	0,0000	0,1575	0,1575	0,0067
R_256	LW_A	0,0538	0,0231	0,0000	0,0976	0,0976	0,0045
	LW_B	0,0063	0,0128	0,0000	0,0546	0,0546	0,0024
R_512	LW_A	0,0000	0,0000	0,0000	0,0000	0,0000	0,0000
	LW_B	0,0000	0,0000	0,0000	0,0000	0,0000	0,0000
NonHBD	LW_A	0,7933	0,0246	0,7455	0,8520	0,1065	0,0048
	LW_B	0,7114	0,0303	0,6666	0,7797	0,1131	0,0056
